# Hyperdimensional computing with holographic and adaptive encoder

**DOI:** 10.3389/frai.2024.1371988

**Published:** 2024-04-09

**Authors:** Alejandro Hernández-Cano, Yang Ni, Zhuowen Zou, Ali Zakeri, Mohsen Imani

**Affiliations:** ^1^Department of Computer Science, École polytechnique fédérale de Lausanne (EPFL), Lausanne, Switzerland; ^2^Department of Computer Science, University of California, Irvine, Irvine, CA, United States

**Keywords:** brain-inspired computing, hyperdimensional computing, holographic representation, vector function architecture, efficient machine learning

## Abstract

**Introduction:**

Brain-inspired computing has become an emerging field, where a growing number of works focus on developing algorithms that bring machine learning closer to human brains at the functional level. As one of the promising directions, Hyperdimensional Computing (HDC) is centered around the idea of having holographic and high-dimensional representation as the neural activities in our brains. Such representation is the fundamental enabler for the efficiency and robustness of HDC. However, existing HDC-based algorithms suffer from limitations within the encoder. To some extent, they all rely on manually selected encoders, meaning that the resulting representation is never adapted to the tasks at hand.

**Methods:**

In this paper, we propose FLASH, a novel hyperdimensional learning method that incorporates an adaptive and learnable encoder design, aiming at better overall learning performance while maintaining good properties of HDC representation. Current HDC encoders leverage Random Fourier Features (RFF) for kernel correspondence and enable locality-preserving encoding. We propose to learn the encoder matrix distribution via gradient descent and effectively adapt the kernel for a more suitable HDC encoding.

**Results:**

Our experiments on various regression datasets show that tuning the HDC encoder can significantly boost the accuracy, surpassing the current HDC-based algorithm and providing faster inference than other baselines, including RFF-based kernel ridge regression.

**Discussion:**

The results indicate the importance of an adaptive encoder and customized high-dimensional representation in HDC.

## 1 Introduction

The human brain remains the most sophisticated yet effective learning module ever. Running on similar power of light bulbs, our brains are in charge of almost every learning and reasoning task in daily life with particularly great sample efficiency and fault tolerance. On the contrary, many widely-applied Machine Learning (ML) algorithms fail to be comparable in efficiency and robustness, despite their prolific advancement in accomplishing practical tasks.

Therefore, research in biological vision, cognitive psychology, and neuroscience has given rise to key concepts behind an emerging field, i.e., brain-inspired computing. In this field, several novel computing paradigms have been developed during the last few years that are either biologically plausible or closer to human brains at the functional level (Roy et al., 2019; Karunaratne et al., 2020). In particular, HyperDimensional Computing (HDC) mimics human brain functionalities when learning and reasoning in high-dimensional spaces (i.e., the hyperspace in HDC), which is motivated by the observation that the human brain operates on high-dimensional neural representations. Similarly in the brain-inspired HDC, a high-dimensional vector-based representation is designed to represent different atomic concepts such as letters, objects, sensor readings, and general features. Typically, an HDC encoder will encode inputs from the original lower-dimensional space to very high-dimensional vectors (i.e., hypervectors in HDC) with several thousand dimensions. Centered on the hypervectors, HDC is also capable of describing the location of objects, their relations, and the structured combination of several individual concepts through a set of HDC operations designed for hypervectors (more details in Section 2).

As the basic building block of HDC, hypervectors own several unique properties that have been crucial for practical applications, especially in terms of representing and manipulating atomic symbols. Specifically, hypervector representation is (1) *holographic*, that information is distributed evenly across components of the hypervector (Kleyko et al., 2023), (2) *robust*, that hypervectors are extremely noise tolerant as a natural result of hypervector redundancy (Kanerva, 2009; Poduval et al., 2022b; Barkam et al., 2023a), and (3) *simple*, that only lightweight operations are needed to perform learning tasks (Hernandez-Cane et al., 2021; Ni et al., 2022b). In addition, the ability for hypervectors to operate symbolically through simple arithmetic has granted HDC the ability to perform cognitive tasks in a transparent and compositional way, e.g., memorization, learning, and association (Poduval et al., 2022a; Hersche et al., 2023). Given the importance of the properties aforementioned, most HDC frameworks have a dedicated and specially designed HDC encoder for mapping original inputs to corresponding hypervectors. The quality of encoded hyperdimensional representations can be decisive for performance in learning and cognitive tasks.

While the HDC encoder has had many variants (Rachkovskij, 2015; Kleyko et al., 2018, 2021; Imani et al., 2019; Frady et al., 2021), most of them innovate on the encoding scheme, i.e., the way symbolically different entities are encoded together. One common example is Position-ID encoding (Thomas et al., 2020): each feature is assigned a (key) hypervector representing its position in the vector, and the value of the feature is quantized to a set of discrete levels and assigned the corresponding (level or value) hypervector. The representation of a feature vector is thus a bundling of several binding key-value pairs. Despite the success of mentioned encoding, the quality of HDC representation of atomic hypervectors is ambiguous: their design is barely discussed due to the already competitive richness in representation (Park and Sandberg, 1991) and performance in practice (Ge and Parhi, 2020). In the case of Position-ID encoding, for example, key hypervectors are assumed to be independent of each other, while value hypervectors preserve a discrete linear similarity with each other. Such manually selected similarity metrics, linear mapping, and discrete atomic compositions naturally lack flexibility. Recognizing this gap, we ask in this paper a fundamental question in improving HDC learning: how can we generate good HDC representation for atomic data? And also, how can we create an encoding scheme that adapts to the problem at hand?

Recent research proposes Vector Function Architecture (Kleyko et al., 2021; Frady et al., 2022) (VFA) that provides a general approach for better representation of continuous data and functions in the hyperspace. Its encoder, instead of presetting discrete levels of similarity for each feature in the original space, directly targets a meaningful similarity in the whole hyperspace such as the Gaussian radial basis function. To do so, VFA relies on fractional power encoding and Random Fourier Features (Rahimi and Recht, 2007) (RFF) parameterized by a high-dimensional encoding matrix through a predefined random distribution. The resulting hyperspace then holds a high-dimensional and non-linear representation that maintains the distance relationship in much finer granularity. We notice that HDC representation quality relies heavily on the choice of hyperspace mapping and similarity metric, which are manifested directly via the distribution from which every component of the encoding matrix is sampled. Recognizing this connection, we expect that selecting a distribution well-adapted to the task will essentially enhance the quality of the HDC encoder as well as learning performance.

In this paper, we bring FLASH, to the best of our knowledge, the first HDC representation that is Fast, Learnable, Adaptive, and Stays Holographic. FLASH leads to an innovative hyperdimensional regression algorithm featuring an optimizable HDC encoder. Unlike all the previous algorithms that limit themselves to either prefixed atomic hypervectors or static encoding mechanisms, our method (1) generates atomic hypervectors that truly adapt to the training data at hand, (2) efficiently optimizes the HDC representation for downstream tasks, and (3) maintains the major benefits of HDC, i.e., holographic representation. We take inspiration from the prior VFA work and propose a novel mechanism to enhance the representation in hyperspace by finding the optimal distribution from which the random matrix is drawn. Moreover, this approach does not require us to use explicitly the kernel function nor the probability density, nor to perform expensive Fourier transforms. This allows the encoding process of FLASH to be as efficient as the static one with the exception of a one-time overhead for optimization.

Our experimental results show that FLASH is about 5.5 × faster in inference than RFF-based ridge regression while providing comparable or better accuracy. We also test a variant called “Accurate FLASH” that is optimized for accuracy, and this approach consistently outperforms other ML baselines, including the previous state-of-the-art HDC regression algorithm (Hernández-Cano et al., 2021) based on VFA. At the same time, we observe a linear increase in our approach with respect to the number of samples in the dataset, making this proposal particularly well-suited for large-scale data.

The rest of this article is organized as follows. In the “HDC Background” section, the basics of HDC are described. And the prior arts VFA-based hyperdimensional regression algorithm is analyzed in the “Regression” section. Our proposed FLASH is formulated in the “Main Methods” section. The “Experimental Results” section presents results for experiments carried out on multiple regression datasets. Finally, the “Conclusion” section concludes this article.

## 2 Related works

In the past few years, prior HDC research works have applied the brain-like functionalities of HDC to diverse applications, for example, outlier detection (Wang et al., 2022), biosignal processing (Rahimi et al., 2020; Ni et al., 2022c; Pale et al., 2022), speech recognition (Hernandez-Cane et al., 2021), and gesture recognition (Rahimi et al., 2016). Apart from classification learning tasks, it has also been applied to genomic sequencing (Zou et al., 2022; Barkam et al., 2023b), nonlinear regression (Hernández-Cano et al., 2021; Ni et al., 2023b), reinforcement learning (Chen et al., 2022; Issa et al., 2022; Ni et al., 2022a, 2023a), and graph reasoning (Poduval et al., 2022a; Chen et al., 2023). With or without hardware acceleration, these HDC algorithms bring a significant efficiency benefit to each application, facilitating online training, few-shot learning, and edge-friendly operation. However, their performance is inevitably limited by a poorly-optimized encoding process. The mapping to hyperspace is either manually devised for a specific task or directly reuses a fixed design such as VFA (Rahimi et al., 2020; Hernández-Cano et al., 2021). In this paper, we focus on improving the HDC encoder design and thus learning performance by proposing a novel encoder that is optimizable and adaptive.

## 3 Regression with vector function architecture

In this section, we first briefly revisit the hyperdimensional encoding technique proposed in VFA. As mentioned in the introduction, the VFA encoding mounts to a well-defined and continuous mapping to hyperspace. We then discuss its usage in the current state-of-the-art HDC regression algorithm and point out the limitation of this method due to the static encoding.

### 3.1 Hyperdimensional encoding in VFA

As a symbolic paradigm, many HDC algorithms operate on a set of dissimilar atomic hypervectors that are randomly generated and near-orthogonal, assuming that symbols are not related at all. However, the assumption will not always be appropriate in practical tasks. Therefore, we have seen HDC algorithms, when handling bio-signals and images, explicitly manipulate the similarity among atomic hypervectors such as maintaining a discrete set of similarity levels. However, the manual assignment of these hypervectors can be problematic, which inevitably causes information loss during quantization, not to mention that such an arbitrarily assumed similarity relationship may not be helpful for learning.

To explain how the VFA encoding captures the relation between data, we note that this encoding coincides with the Random Fourier Features (RFF) encoding, an efficient approximation of kernel methods. The following theorem by Salomon Bochner (1899–1982) serves as the foundation for this well-defined similarity relationship (Rudin, 2017).

** Theorem 1 (Bochner)**. For any continuous shift-invariant and positive definite kernel $K(x_{1}-x_{2}):{\mathbb{R}}^{M}\to \mathbb{R}$, there must exist a non-negative measure *p*(**ω**) such that *K* is the Fourier transform of a non-negative measure *p*(**ω**). Additionally, if *K* is properly scaled, *p*(**ω**) is a proper probability measure.

The proof of this theorem is provided in Rudin (2017). If we assume ζ(**x**) = *e*^*j***ω**^^*T*^**x**, then Theorem 1 leads to the following equation: $K(x_{1}-x_{2})=\int_{{\mathbb{R}}^{M}}p(\omega )e^{j\omega^{T}(x_{1}-x_{2})}=E_{\omega }\left[\zeta (x_{1})\bar{\zeta (x_{2})}\right]$. This means that, with the correspondence between kernel *K* and measure *p*(**x**), we can transform original inputs to a space where dot products are unbiased estimates of kernel similarities. In other words, there exist sequences of transformations $\phi_{D}:{\mathbb{R}}^{M}\to {\text{C}}^{D}$ such that $\;\phi_{D}{\left(x_{1}\right)}^{T}\phi_{D}(x_{2})$ converges uniformly to the given kernel *K*(**x_1_**−**x_2_**):


(1) $$\phi_{D}{\left(x_{1}\right)}^{T}\bar{\phi_{D}(x_{2})}\overset{large\;D}{\to }K(x_{1}-x_{2})$$

Rahimi and Recht (2007) proposed an alternative set of Random Fourier Features (RFF) such that the components of the encoded vectors are real and the kernel approximation converges equally fast. To construct a real-valued RFF, we can leverage this high-dimensional mapping for HDC encoder as the following:


(2) $$\begin{array}{l} \phi_{D}\left(\text{x};\text{Ω},\text{b}\right)=\sqrt{\frac{2}{D}}cos.\left(\text{Ω}\text{x}+\text{b}\right) \end{array}$$

where cos. represents the element-wise cosine function, **Ω**∈ℝ^*D*×*M*^ is a randomly generated encoding matrix, and **b**∈ℝ^*D*^ is the offset hypervector. Row vectors in **Ω** are generated by drawing *D* i.i.d. samples **ω**_1_, …, **ω**_*D*_ from *p*(**ω**) and elements in *b* are sampled from U[0,2π]. The random distribution *p* is selected through Theorem 1 given a preferred kernel *K*(Δ). In other words, the probability density is calculated with a Fourier transform: $p\left(\text{ω}\right)=\frac{1}{2\pi }\underset{{\mathbb{R}}^{M}}{\int }\exp\left(-i{\text{ω}}^{T}\Delta \right)K\left(\Delta \right)d\Delta$. For example, previous HDC work (Hernández-Cano et al., 2021) uses Normal distribution N(0,1) for *p*(**ω**) since it wants to approximate the Gaussian RBF kernel.

There are key lessons from the theory of VFA encoding discussed above:

1. HDC encoding as in Equation (2) incorporates a high-dimensional non-linear mapping through the cosine activation.
2. Due to RFF, it supports a meaningful similarity metric in hyperspace without quantizing individual features or generating ambiguous correlated base hypervectors.
3. By Bochner's theorem, there is a correspondence between kernel *K* and measure *p*(**x**). This implies that we can leverage the measure for the estimation of kernel similarities.

While the current VFA method brings many benefits, the biggest drawback of this method is that ϕ_*D*_(**x**) is essentially a static mapping, which makes the encoding less adaptive. Our work aims to leverage the insight from Bochner's theorem to learn the kernel adaptively through its random Fourier features.

### 3.2 Regression on a static HDC encoder

Ideally, we expect the HDC encoder to provide a useful high-dimensional representation that helps separate the data points for classification or linearize the inherent non-linear regression tasks. Particularly in hyperdimensional regression, we are interested in finding the best hypervector **w**∈ℝ^*D*^ such that the linear regression after encoding *y*(**x**) = ϕ(**x**)^*T*^**w** are, in average across the training set, as close as possible to the true labels in terms of ℓ_2_ norm. Additionally, we introduce an ℓ_2_ regularization coefficient λ to get more stable estimators. Thus the loss function is the dampened least squares, which can be expressed as


(3) $$\begin{array}{l} L_{R}\left(\text{w}\right):={||\text{Z}\text{w}-\text{y}||}^{2}+\lambda {||\text{w}||}^{2}, \end{array}$$

where $\text{y}={\left(y_{1},y_{2},\ldots ,y_{N}\right)}^{N}$ are the known response variables, and **Z** = Φ(**X**; **Ω**, **b**)∈ℝ^*N*×*D*^ are the encoded hypervectors of the input data **x**_1_, …, **x**_*N*_:


(4) $$\begin{array}{l} \Phi \left(\text{x};\text{Ω},\text{b}\right)=\left(\begin{array}{c} \phi {\left({\text{x}}_{1};\text{Ω},\text{b}\right)}^{T}\\ \vdots \\ \phi {\left({\text{x}}_{N};\text{Ω},\text{b}\right)}^{T} \end{array}\right)=\sqrt{\frac{2}{D}}cos.\left(\text{x}{\text{Ω}}^{T}+\text{b}\right). \end{array}$$

In previous approaches for HDC regression (Hernández-Cano et al., 2021; Kleyko et al., 2021), the model hypervector **w** is learned in an iterative fashion, where hypervectors are bundled together guided by regression errors. However, they face issues with proper hyperparameter selection to achieve the highest prediction quality. On the other hand, the loss function in Equation (3) has a known minimizer $\hat{\text{w}}$ which is known as the ridge estimator:


(5) $$\begin{array}{l} \hat{\text{w}}={\left({\text{Z}}^{T}\text{Z}+\lambda \text{I}\right)}^{-1}{\text{Z}}^{T}\text{y}. \end{array}$$

In this paper, we leverage this statistical approach to obtain better stability during learning and more direct parameter tuning.

As we mentioned in the previous section, the encoding in VFA, the closed solution in Equation (5) has an assumption that the regression problem on **Z** is linearly solvable, as the result of mapping to hyperspace. However, for an arbitrary regression task, it is very likely that the static VFA encoding (due to the fixed *K*(Δ) and *p*(**ω**)) becomes sub-optimal. This work looks to address this problem by presenting an adaptive HDC encoder design.

## 4 Main methods

In this paper, we proposeFLASH, a way to learn a good encoding function ϕ(**x**) before solving the regression task in hyperdimensional space.

**Figure 1:**
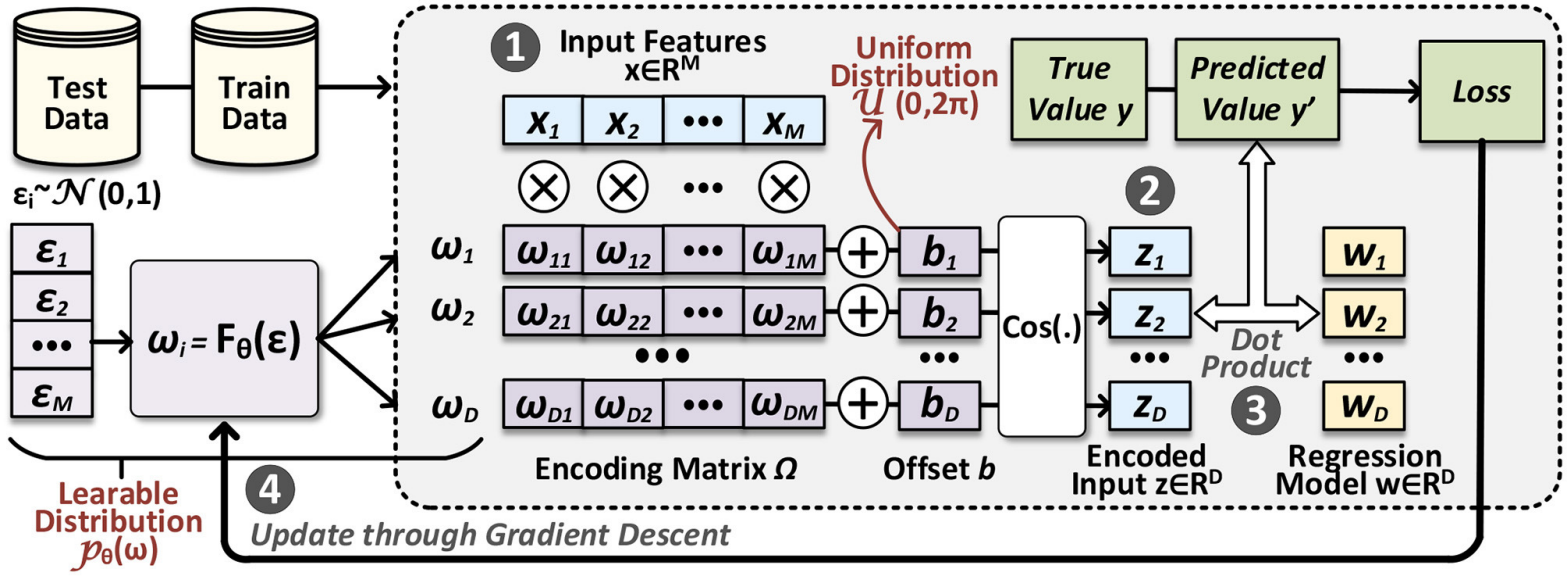
Overview of our proposed FLASH: the area with shade represents the inference process; the rest modules are related to adapting the HDC encoder, where we learn the distribution of the encoding matrix.

In Figure 1, we present an outline of our proposed FLASH, including both HDC inference and encoder learning processes. In the inference process, we start from a query data point **x**∈ℝ^*M*^ in the original space ❶, which is then passed through the encoding module ❷ to obtain the encoded data point **z**∈ℝ^*D*^ in the hyperspace. Once we have this query hypervector, getting the prediction ŷ reduces to perform dot product with the regression hypervector ❸. The overall inference process depicted here is similar to VFA-based regression; what distinguishes FLASH from prior algorithms is that the encoding module (**Ω** matrix, specifically) is obtained through a parameterized distribution *p*_θ_. During the encoder learning ❹, parameters in *p*_θ_ are updated given the feedback from the regression loss defined in Equation (3). In the following sections, we will introduce how to sample from this parameterized distribution and learn its parameters.

### 4.1 Generating the encoding matrix

Care must be taken when designing the HDC encoder, as it needs to ensure that the appealing properties of the hypervectors are sustained. To ensure the holographic representation, we require the randomized instantiation of the encoder, and thus we cannot directly perform gradient descent upon an instantiated encoding matrix, as it may destroy both: information about the data may be distributed locally and partially in the output vector, and the trained encoder have minimum randomization.

To circumvent this, we take inspiration from the VFA work that highlights the encoder-kernel correspondence and the importance of selecting a proper distribution *p*(**ω**). Recall from Section 3.1 that there is a correspondence between kernel *K* and measure *p*(**x**). In addition, it induces families of encoders {_ϕ_*D*_}*D*∈ℕ_ parameterized by the samples from the distribution such that the inner product between the encoded vectors approximates the kernel. This approximation improves increasingly well with the dimension of the encoder (codomain). By learning the distribution from which we sample the encoding matrix, we will be able to construct an adaptive HDC encoder that provides a more suitable hyperdimensional representation, adding to existing appealing HDC properties.

In FLASH, we define a parametric family of functions $F=\left\{f_{\text{θ}}:f_{\text{θ}}:{\mathbb{R}}^{M}\to {\mathbb{R}}^{M},\text{θ}\in \text{Θ}\right\}$, which will be referred to as generators. Upon receiving random inputs, it can be used to sample random vectors **ω**_1_, …, **ω**_*D*_ required in the encoding function (Equation 4). Compared to parameterized distributions such as the Gaussian reparameterization approach, this approach encapsulates a more expressive family of distributions. Because of Bochner's theorem, exploring F is equivalent to exploring a corresponding set of continuous shift-invariant kernels, and thus the encoding family we consider is expected to be rich as long as F is.

Inherited from the RFF methods, a key benefit of this arrangement is that FLASH encoding does not require us to use explicitly the kernel *K*(Δ) function, nor the probability density *p*(**ω**), nor to perform expensive Fourier transforms. Our goal is to find f∈F that, with a high probability, gives the optimal or near-optimal encoding matrix of solving the regression problem at hand; this ensures the quality and robustness of the encoder that it generates.

### 4.2 Learning the encoder matrix distribution

To learn the distribution efficiently, we restrict F to be a family of $f_{\text{θ}}:{\mathbb{R}}^{M}\to {\mathbb{R}}^{M}$ differentiable neural networks, i.e., the network input size equals the output size. To sample **ω**s using *f*_**θ**_, we first draw a random vector ϵ~N(0,I) as the input, and then obtain a transformed random vector using **ω** = *f*_**θ**_(**ϵ**). Sampling *D* i.i.d. random noises and passing them through the generator can give us a matrix of base hypervectors (i.e., **Ω**), which can be used to perform the encoding. This can be understood as a generalized reparameterization, where we learn a surrogate function *f*_**θ**_(**ϵ**) instead. Note that we chose to sample noise vectors from the standard normal distribution for convenience, but different choices can be made as well. This architecture gives us a very rich family of generators F, which are cheap to evaluate and cheap to train, as we will see in the next few sections. In addition, because **ϵ** is a random vector, **ω** = *f*_**θ**_(**ϵ**) is one too, which means that there exists a probability density (or mass) function *p*_**θ**_(**ω**) for each generator *f*_**θ**_.

In FLASH, we aim to maximize encoder performance in generating a good HDC representation of our data for the regression task. In particular, we opt to evaluate the learned encoder using the loss function proposed in Equation (3). However, because random sampling is involved at the moment of generating the encoding, we chose to minimize the expected value instead. Note that this expected value is taken with respect to all the possible encoding **Φ**, whose encoding matrix **Ω** and offset hypervectors **b** are randomly sampled. Thus, in Equation 6, we seek to find the parameters in *f*_**θ**_ that minimize the following loss term for adapting the HDC encoder:


(6) $$\begin{array}{l} L_{E}\left(\text{θ}\right)=\underset{\text{Ω},\text{b}}{E}\left[\underset{\text{w}\in {\mathbb{R}}^{D}}{\min}L_{R}\left(\text{w}\right)\right] \end{array}$$

where $L_{R}\left(\text{w}\right)$ is the regression loss defined in Equation (3). In Equation 7, using the ridge estimator $\hat{\text{w}}$ and the law of the unconscious statistician, we can expand the previous expectation term to:


(7) $$\begin{array}{l} \underset{\Omega ,b}{\text{E}}\left[{ℒ}_{R}(\hat{w})\right]=\underset{\begin{array}{c} \omega_{1}..\omega_{D}\sim p_{\theta }\\ b_{1}..b_{D}\sim U \end{array}}{\text{E}}\left[\|\Phi (X;\Omega ,b)\hat{w}-y{\|}^{2}+\|\hat{w}{\|}^{2}\right]\\ \;=\underset{\begin{array}{c} \epsilon_{1}..\epsilon_{D}\sim N\\ b_{1}..b_{D}\sim U \end{array}}{\text{E}}\left[\|\Phi (X;f_{\theta }(E),b)\hat{w}-y{\|}^{2}+\|\hat{w}{\|}^{2}\right] \end{array}$$

where **E** is the matrix containing the *D* random vectors **ϵ**_1_, …, **ϵ**_*D*_. Thus, we can obtain an unbiased estimator of the encoding loss using a simple Monte Carlo estimator with a single sample. That is, if we sample **E** and **b** from their respective distributions, we obtain an unbiased estimator of $L_{E}\left(\text{θ}\right)$:


(8) $$\begin{array}{l} {\hat{L}}_{E}\left(\text{θ}\right)={||\Phi \left(\text{x};f_{\text{θ}}\left(\text{E}\right),\text{b}\right)\hat{\text{w}}-\text{y}||}^{2}+{||\hat{\text{w}}||}^{2} \end{array}$$

Note that it is possible to sample multiple noise matrices **E**_1_, **E**_2_, … to lower the variance of the estimator, but in order to accelerate the computation we don't explore this alternative.

### 4.3 Adapt the encoder via generator training

We explore the parameter space of the generator *f*_**θ**_ using stochastic gradient descent, where $\text{θ}\leftarrow \text{θ}-\eta \nabla_{\text{θ}}{\hat{L}}_{E}\left(\text{θ}\right)$. Its easy to show that $\nabla_{\text{θ}}\hat{L}\left(\text{θ}\right)$ is an unbiased estimator of the true gradient $\nabla_{\text{θ}}L\left(\text{θ}\right)$ because the expectation in $L_{E}$ does not depend on the parameters **θ**. It is important to note that ${\hat{L}}_{E}$ is differentiable with respect to its parameters **θ**. Indeed, every operation shown in Equation (8) is well behaved: **Ω** = *f*_**θ**_(**E**) is clearly differentiable, and so is **Z** = Φ(**X**; **Ω**, **b**) because $\varphi \left(\text{x};\text{Ω},\text{b}\right)=\sqrt{\frac{2}{D}}cos.\left(\text{Ω}\text{x}+\text{b}\right)$.

Until here, we have covered how to parameterize and learn the sampling distribution. This equips FLASH with an encoding module well-adapted. Still, people may wonder if learning the encoding matrix **Ω** and offset **b** through gradient descent is a good alternative. We acknowledge that this might be a more direct measure, however, it will jeopardize the holographic property of HDC since it cannot guarantee that the encoding matrix is i.i.d. This means that the information in the encoded hypervector is no longer evenly distributed, and errors or noise in the encoding process will lead to higher performance loss due to the lack of hyperdimensional redundancy. Our proposed measure will ensure that FLASH will maintain the holographic HDC representation after tuning the encoder.

### 4.4 Balance the cost in training

The training in FLASH is a two-stage process where we first learn the generator *f*_**θ**_(**ϵ**), i.e., in place of sampling from *p*_**θ**_(**ω**)). Based on the first training stage, we then perform the model training that gives the regression hypervector **w**. In the second stage, the encoder will be generated using *f*_**θ**_ and remain static as it has been optimized. Notice that the dimensionality *D* can be the same or different in these two training stages. As mentioned in the previous section, our optimization method for generator training is well-defined; but in practice, it can be further approximated to obtain faster convergence. Several algorithms have been widely used to accelerate the computation of ridge estimator (Paige and Saunders, 1982; Defazio et al., 2014). However, computing $L_{R}\left(\text{θ}\right)$ at every iteration for encoder learning ends up adding up the overhead. Recall that in prior HDC algorithms (Hernandez-Cane et al., 2021; Hernández-Cano et al., 2021; Ni et al., 2022a), *D* is supposed to be a high dimensionality such that model hypervectors have a larger capacity. In FLASH, we instead propose to decouple the high dimensionality requirement from the encoder/generator training since the generator *f*_**θ**_ itself operates in ℝ^*M*^. When training *f*_**θ**_, we encode data to ℝ^*D*^′ instead, where *D*′ < *D* in order to accelerate the process. As for the regression process, we use a slightly larger dimensionality *D* for better regression accuracy. In fact, adapting the HDC encoder at first will also lower the requirement for model hypervector dimensionality *D* and thus reduce the training cost. Our results in the experiment show that FLASH, with a lower dimensionality, has comparable regression quality to the prior HDC method.

### 4.5 Time complexity

In this section, we discuss the time complexity of training our proposed method. Below we describe at a high level the steps required in our approach.

1. Train the surrogate sampling function *f*_**θ**_.

(a) Encode data to *D*′-dimensional space.
(b) Compute the loss $L_{E}\left(\text{θ}\right)$.
(c) Compute the gradient and update the parameters in *f*_**θ**_.
(d) Iterate this process until convergence.

2. Generate encoding matrix **Ω** using *f*_**θ**_.
3. Encode data to *D*-dimensional with the adapted HDC encoder.
4. Learn the regressing hypervector **w**.

In the first step, the overhead of computing $L_{E}$ is considered minor as we encode data to *D*′-dimensional space, limiting the cost of computing the estimator $\hat{\text{w}}$. Generating random bases **ω**_*i*_ = *f*_**θ**_(**ε**_*i*_) requires *D* forward passes of *f*_**θ**_, which will give a hyperdimensional mapping.

Encoding the data $\text{Z}=\Phi \left(\text{x};\text{Ω},\text{b}\right)=\sqrt{\frac{2}{D}}cos.\left(\text{x}{\text{Ω}}^{T}+\text{b}\right)$, requires O(NMD) operations, corresponding to the asymptotic of the most taxing operation - matrix multiplication of the *N*×*M* matrix **X** with a *M*×*D* matrix **Ω**^*T*^, where *N* is the number of samples and *M* the number of features in original space.

The last step, computing **w** = (**Z**^*T*^**Z**+λ**I**)−1**Z**^*T*^**y** is, according to experimental evaluation, the most time-consuming step in our design. The theoretical time complexity is dominated by the **Z**^*T*^**Z** multiplication and the inverse operation, requiring $O\left(ND^{2}\right)$ and $O\left(D^{3}\right)$ operations, respectively. Thankfully, the dampened linear least squares loss, Equation 3), has been heavily studied and several algorithms that approximate its solution exist such as LSQR (Paige and Saunders, 1982). Moreover, the experimental evaluation suggests that with relatively small values of *D* (500 ≤ *D* ≤ 2000) we can obtain very accurate predictions (as shown in Figure 5); with this configuration, we observe linear training time in the number of samples.

### 4.6 Formal derivation of encoding loss

In this section, we show that minimizing $L_{E}$ with respect to **θ** is equivalent to maximizing a lower bound of the log-likelihood of the posterior distribution with joint parameters (**θ**, **w**).

Recall that given a dataset of independent observations D, random parameters θ∈Θ with prior distribution θ~*p*(θ) the posterior distribution is p(θ∣D), which by Bayes' Theorem can be expressed as p(θ∣D)∝p(D∣θ)p(θ), where p(D∣θ) is called the likelihood.

In regression analysis, we assume the relation **y** = **Xw**+**ε**, where **ε** is a random unobserved noise. Ordinary least squares regression sets the likelihood to be normally distributed $\text{y}|\text{w}\sim N\left(\text{x}\text{w},\sigma^{2}\text{I}\right)$. As shown in Equation 9, maximizing the log-likelihood is equivalent to minimizing the squared error loss:


(9) $$\begin{array}{l} \underset{w}{\max}p(y|w)=\underset{w}{\max}\ln p(y|w)=\underset{w}{\max}\ln(\frac{1}{Z}\exp(-\frac{1}{2}{\left(y-Xw\right)}^{T}{\left(\sigma^{2}I\right)}^{-1}(y-Xw)))\\ \;=\underset{w}{\max}\{\ln\exp(-\frac{1}{2}{\left(y-Xw\right)}^{T}{\left(\sigma^{2}I\right)}^{-1}(y-Xw))-\ln Z\}\\ \;=\underset{w}{\min}\frac{1}{2\sigma^{2}}{\left(y-Xw\right)}^{T}(y-Xw)=\underset{w}{\min}\|y-Xw{\|}^{2}. \end{array}$$

In order to work in the high-dimensional space, we must add the encoding to the equation, or in this case the generator parameters **θ**. We instead assume the relation **y** = **Zw**+**ε**, where **Z** = Φ(**X**; **Ω**, **b**). Because the regression coefficients depend on **Ω** and **b**, we use the conditional likelihood $\text{y}|\text{Ω},\text{b},\text{w}\sim N\left(\text{Z}\text{w},\sigma^{2}\text{I}\right)$. We add the independence assumption between **θ** and **b**. In Equation 10, the maximum log posterior is shown to be bounded using Jensen's inequality:


(10) $$\begin{array}{l} \underset{\theta ,w}{\max}\left\{\ln p(\theta ,w|y)\right\}=\underset{\theta ,w}{\max}\left\{\ln\left(p(y|\theta ,w)p(\theta )p(w)\right)\right\}\\ =\underset{\theta ,w}{\max}\left\{\ln\;\underset{\begin{array}{c} \omega_{1}..\omega_{D}\sim p_{\theta }\\ b_{1}..b_{D}\sim U \end{array}}{\text{E}}\left[p(y|\Omega ,b,w)p(\theta )p(w)\right]\right\}\\ \geq \underset{\theta ,w}{\max}\left\{\underset{{\;}_{\begin{array}{c} \omega_{1}..\omega_{D}\sim p_{\theta }\\ b_{1}..b_{D}\sim U \end{array}}}{\text{E}}\left[\underset{-{ℒ}_{R}(w)}{\underset{︸}{\ln p(y|\Omega ,b,w)+\ln p(w)}}+\ln p(\theta )\right]\right\}\\ =\underset{\theta }{\min}\underset{w}{\min}\left\{\underset{{\;}_{\begin{array}{c} \omega_{1}..\omega_{D}\sim p_{\theta }\\ b_{1}..b_{D}\sim U \end{array}}}{\text{E}}\left[{ℒ}_{R}(w)-\ln p(\theta )\right]\right\}\\ =\underset{\theta }{\min}\left\{\underset{{\;}_{\begin{array}{c} \omega_{1}..\omega_{D}\sim p_{\theta }\\ b_{1}..b_{D}\sim U \end{array}}}{\text{E}}\left[{ℒ}_{R}(\hat{w})+R(\theta )\right]\right\}=\underset{\theta }{\min}{ℒ}_{E}(\theta ). \end{array}$$

## 5 Experimental results

### 5.1 Experimental settings

We implement the proposed design using Python on the Intel Core i7-12700K CPU platform. The core process of adapting the encoder is implemented using Pytorch, and the regression process is based on the implementation provided by Scikit-Learn. We evaluate the accuracy of our design on several practical regression datasets listed in Table 1, with up to 20,000 samples and 80 features. Table 2 describes the baseline models used to compare with our design, including ridge regression that also leverages RFF approximation and the previous state-of-the-art HDC-based regression algorithm RegHD. During the experiments, we test two settings for our design (FLASH and A-FLASH), slightly different in model size and dimensionality. The name of the second setting stands for “Accurate FLASH,” which has larger dimensionality and model size. In Table 3, we provide the hyperparameters used for the different regression models in our experiments.

**Table 1:** Various regression datasets available on OpenML (Vanschoren et al., 2013).

**Dataset**	***N***	***M***	**Description**
kin8nm	8,192	9	Forward kinematics of an 8 link robot arm
MiamiHousing2016	13,932	17	Sale price of houses
pol	15,000	49	Telecommunication problem
Houses	20,640	9	Predict house value
Superconduct	21,263	82	Predict critical temperature of superconductors

N, Number of samples; M, number of features.

**Table 2:** Regression models used in experiments: the last two are our proposed design, and the rest are baseline methods.

**Model**	**Description**
SVR	Support vector regression with RBF kernel
Kernel Ridge	Analytical solution of kernel regression with RBF kernel
Linear Regression	Ordinary least squares
RFF + Ridge	RFF approximation of RBF kernel, followed by ridge regression
RegHD	HDC regression based on VFA (Hernández-Cano et al., 2021)
FLASH	Our design optimized for better inference runtime
A-FLASH	Our design optimized for better regression accuracy

**Table 3:** Hyperparameters used for the regression models.

**Model**	**Hyperparameter**	**Value**
FLASH (A-FLASH)	*D*	500 (resp. 2000)
	*D*′	75 (resp. 250)
	α^†^	0.01 ≤ α ≤ 0.1
	*f*_**θ**_ layers	[32, 32] (resp. [64, 64, 64, 64])
	*f*_**θ**_ activation	tanh
	*f*_**θ**_ learning rate^†^	0.001 ≤ lr ≤ 0.01
*RegHD	*D*	2000
	Number of models	1
	Learning rate	0.035
RFF + Ridge	*D*	2000
*SVR	*C*	0.1 ≤ *C* ≤ 100
	ε	0.01 ≤ ε ≤ 1
	γ	$\frac{1}{\text{n}\text{\_}\text{feature}{\text{s}}^{*}\text{Var}\left(X\right)}$

^†^This hyperparameter was tuned using grid search. ^*^SVR means support vector regression and RegHD is the name of a prior HDC-based regression framework.

### 5.2 Performance on synthetic data

**Figure 2:**
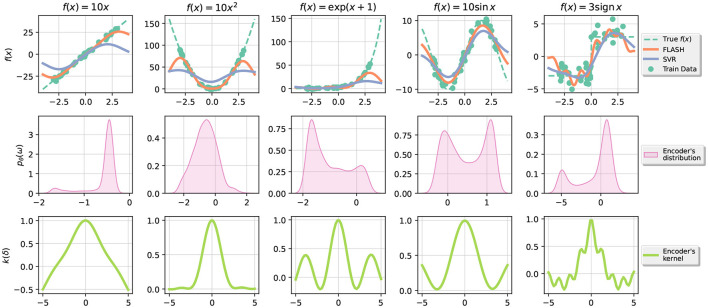
The **top row** corresponds to the data created, in green is the training data, in orange is the prediction of FLASH, and in blue is the prediction of SVR. The **second row** corresponds to the distribution *p*_**θ**_(**ω**) of the random bases in the encoder. The **last row** shows the (approximate) kernel associated with the distribution as in Equation (1).

In this section, we analyze the performance of the proposed design in custom 1D regression problems of the form *y* = *f*(*x*)+ε with ε~N(0,1) and different choices of target function *f*. In Figure 2, we also show the encoder's probability distribution *p*_**θ**_(**ω**) learnt in the process and the actual kernel function *K*(Δ) = E[ϕ(**x**)^*T*^ϕ(**0**)]. For comparison, RBF kernel has associated a Gaussian $N\left(\text{0},\frac{1}{\gamma }\text{I}\right)$ distribution.

From this experiment, we conclude that our optimization proposal for the encoder loss $L_{E}$ works well in practice and the shapes of learned distributions are varied for each dataset. Moreover, we observe that our proposal adapts to different scales in the data making a clear distinction with SVR. For instance, the first predicted function *f*(*x*) = 10*x*^2^, where SVR clearly underperforms where |*x*|>1.1.

### 5.3 Regression quality and efficiency comparison

**Figure 3:**
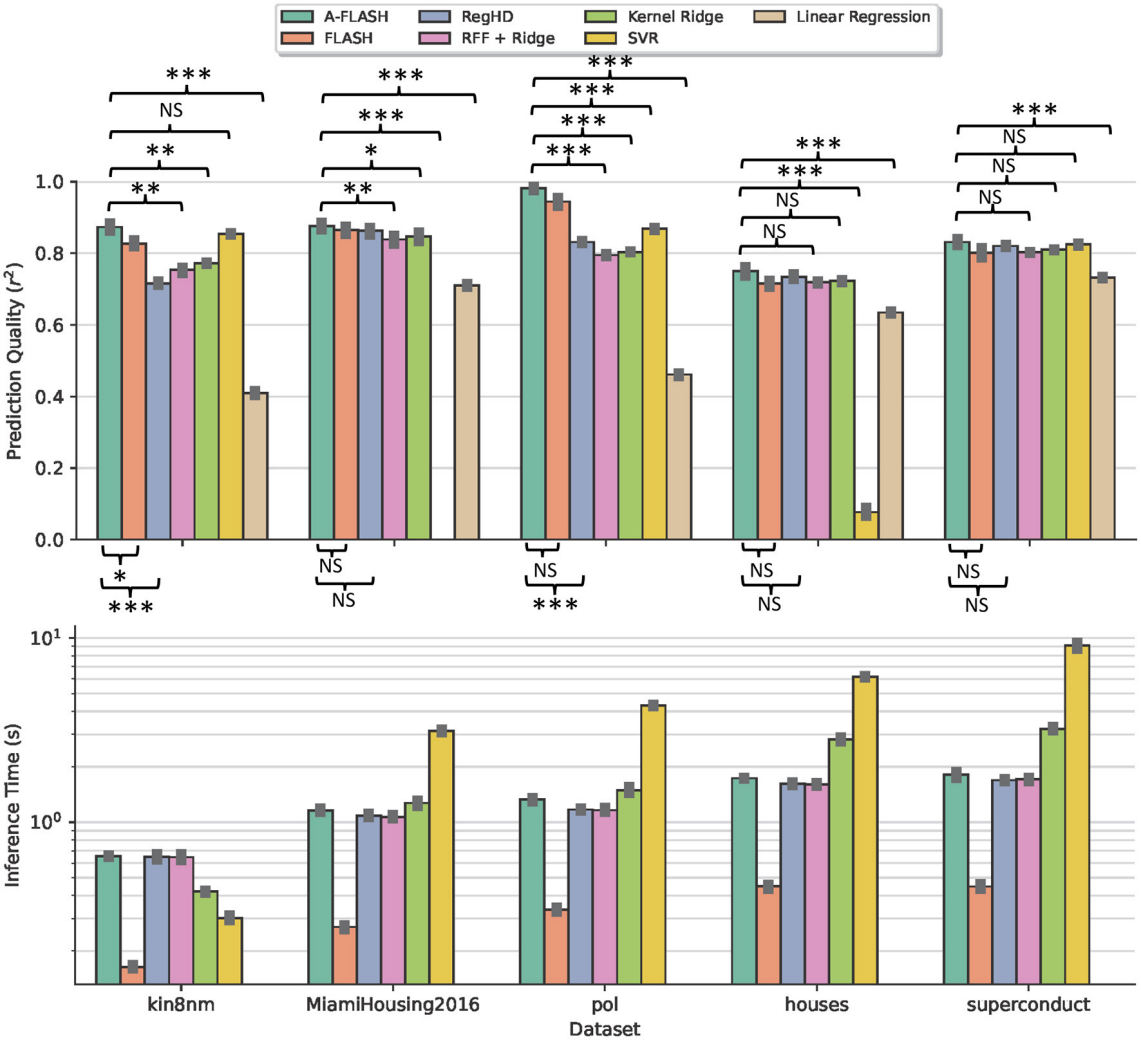
Comparison of our approach against other methods in several datasets. The **top plot** shows the prediction quality using the *r*^2^ metric (larger is better). The **bottom plot** shows the inference time. We exclude the linear regression runtime for better visualization since the value is relatively small. Note that SVR performs poorly on the MiamiHousing dataset even after grid search (thus not visible in the figure) and the log scale is used in the bottom plot. We report the ±95% confidence interval and use the Nadeau and Bengio's corrected *t*-test (Nadeau and Bengio, 1999) for significance in prediction quality comparison: ^***^P < 0.001, ^**^P < 0.01, ^*^P < 0.05; NS, not significant.

In this section, we compare the performance of FLASH (as well as A-FLASH) against several baseline regression algorithms using multiple regression datasets. We perform 5 times repeated 5-fold cross-validation in each dataset and report the average prediction quality, confidence intervals, statistical tests for significance, and runtime taken for each fold. We select the most important hyperparameters in SVR and our design using grid search. Our results are summarized in Figure 3.

We observe that our approach is always comparable in accuracy with other state-of-the-art approaches. The accurate version of our approach (A-FLASH) is consistently ranked at the top. Particularly, because our encoder is learnable and well-adapted, we are generally more accurate than other algorithms leveraging static encoder or fixed kernel. In comparison with the prior HDC-based method, A-FLASH achieves significantly better quality without adding notable overhead for inference. In addition, the fast version of our approach (FLASH) is generally among the fastest models. During inference, it is faster than other baselines, including classical kernel-based approaches such as SVR and Kernel Ridge. This is because our prediction complexity is constant with respect to the number of samples. On average, FLASH is about 3.7 × faster inference than the RegHD, 5.5 × faster than kernel ridge/RFF ridge, and 13.75 × faster than SVR.

### 5.4 Scalability results

**Figure 4:**
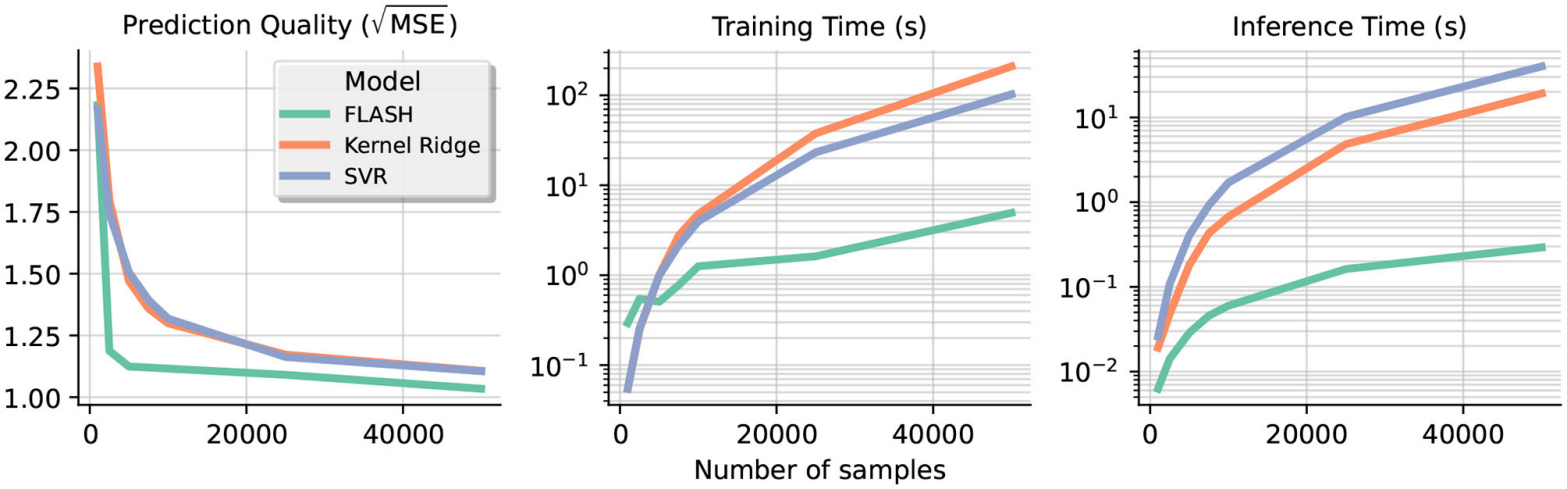
The **left graph plots** the prediction quality (root MSE) against the number of samples in different regression models. The **second and third plot** shows the time taken to train and do inference on a logarithmic scale, respectively. Note the slower growth rate of our approach compared with other kernel approaches.

In this section, we create the Friedman regression datasets (Friedman, 1991) with an increasing number of samples to test the scalability of the proposed algorithm and compare it with other approaches. We observe that our approach is well-suited for large-scale data as we have a linear trend in the time taken to train and also inference time. Meanwhile, the time taken to train classical kernel approaches such as SVR and kernel ridge grows noticeably faster due to their higher computational complexity. Our results are summarized in Figure 4. The leftmost plot shows that our approach is the fastest to achieve high prediction quality even with a small number of samples; in fact, FLASH constantly achieves better accuracy when the training set grows. In terms of inference speed, FLASH is about twice as fast as the other approaches with 5000 training samples, and the gap in between continues to expand.

### 5.5 Impact of dimensionality

**Figure 5:**
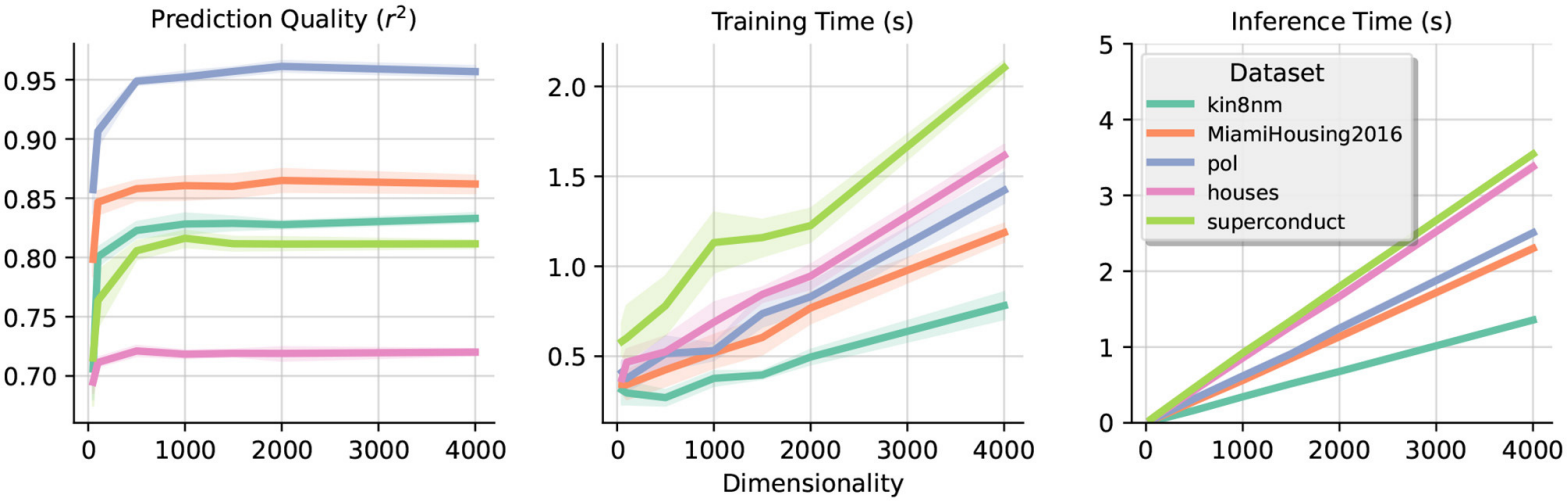
Impact of dimensionality. Each graph shows the trade-off obtained when increasing the dimensionality *D*. The metrics measured are the coefficient of determination (*r*^2^), training time, and inference time, respectively across the three plots. Notice the fast convergence of accuracy even with low dimensionality and the approximately linear scale in the time measured.

In this section, we explore the impact of dimensionality (*D*) in our design for various datasets. Figure 5 displays our results in terms of prediction quality (MSE) and time taken to train the model for different values of *D*. In the section on “Time Complexity,” we derived the time complexity of our approach to be $O\left(ND^{2}\right)$, which is consistent with our experimental results. However, it is worth mentioning that even for relatively small dimensionality (e.g., *D* = 500) the gain of accuracy for further increasing dimensionality is not significant. Thus, even if the theoretical complexity of the approach is large, in practice, we can obtain acceptable results rapidly.

## 6 Conclusion

In this paper, we present a novel HDC algorithm that features an adaptive and learnable encoder design. Unlike previous HDC works that solely focus on the learning of model hypervectors, our work also aims at providing a hyperdimensional representation that is more suitable to current tasks. Instead of learning the encoder directly, we construct a parameterized distribution that helps preserve the holographic property of HDC encoding. The results of several regression tasks show that our proposed algorithm can significantly boost the accuracy, surpassing the existing HDC-based arts and providing lower inference time.

## Data availability statement

Publicly available datasets were analyzed in this study. This data can be found here: https://www.openml.org/search?type=data.

## Author contributions

AH-C: Conceptualization, Data curation, Formal analysis, Investigation, Methodology, Project administration, Software, Supervision, Validation, Visualization, Writing – original draft, Writing – review & editing. YN: Conceptualization, Data curation, Formal analysis, Investigation, Methodology, Software, Validation, Visualization, Writing – original draft, Writing – review & editing. ZZ: Conceptualization, Formal analysis, Investigation, Methodology, Validation, Writing – original draft, Writing – review & editing. AZ: Data curation, Formal analysis, Investigation, Methodology, Validation, Writing – original draft, Writing – review & editing. MI: Conceptualization, Data curation, Formal analysis, Funding acquisition, Investigation, Methodology, Project administration, Resources, Software, Supervision, Validation, Visualization, Writing – original draft, Writing – review & editing.
